# Identification of Immunosuppressive Medication Nonadherence Factors Through a Combined Theory Model in Renal Transplant Recipients: 6–12

**DOI:** 10.3389/fphar.2021.655836

**Published:** 2021-05-26

**Authors:** Pengpeng Zhang, Xiao Zhu, Jin Yan, Jia Liu

**Affiliations:** ^1^ Department of Transplantation, The Third Xiangya Hospital of Central South University, Changsha, China; ^2^ The Nursing Department, the Third Xiangya Hospital of Central South University, Changsha, China; ^3^ The Nursing School of Central South University, Changsha, China

**Keywords:** renal transplantation, immunosuppressive medication nonadherence, theory of planned behavior, health belief model, structural equation

## Abstract

**Background:** Immunosuppressive medication (IM) nonadherence is associated with poor transplant outcomes. Therefore, it is of great importance to identify predictive factors with IM nonadherence. We aimed to improve the predicted capacity of the theory of planned behavior (TPB) by adding health belief model’s (HBM) variables in renal transplant patients (RTPs).

**Methods:** This cross-sectional study distributed questionnaires to patients who had undergone renal transplant and follow-up regularly in the transplant center of Third Xiangya Hospital in China. The self-developed questionnaire collected data in three aspects: general data questionnaire, TPB, HBM-specific questionnaire, and Basel Assessment of Adherence to Immunosuppressive Medications scale.

**Results:** A total of 1,357 of 1,480 patients completed the survey, with a participation rate of 91.69% and IM nonadherence rate of 33.53%. The marital status, household income, preoperative drinking history, the time after transplantation, and religion showed independent predictive factors with IM nonadherence (*p* < 0.05). Strikingly, adding HBM variables to the TPB theory model significantly increased its prediction ability to IM nonadherence (52%). Also, HBM manifested the highest coefficient of effect (−0.620). Particularly, perceived barriers and perceived seriousness, the variables of the HBM model, played a vital influence on medication nonadherence (−0.284 and 0.256).

**Conclusion:** Our study here reveals the first investigation of the combined effects of the TPB and HBM model on IM nonadherence in Chinese RTPs, which could significantly improve the predictive ability of any single model. Meanwhile, future interventions should be conducted to both increase perceived seriousness and reduce perceived barriers for taking IM, which will effectively decrease IM nonadherence rates and improve transplant outcomes.

## Introduction

Since 1954, when the first renal transplant was successfully conducted between twins, a series of sustainable progresses such as refined surgical techniques improved immunosuppressive protocols and optimized perioperative management of transplant patients have been utilized to improve patient and graft survival following the renal transplant (Tonelli et al., 2011). However, the renal transplant community has been now challenged by acute rejection and transplant graft loss because of IM nonadherence (Prihodova et al., 2014). A recent study showed that IM nonadherence could lead to increasing risk of cancer over the long term in renal transplant patients (RTPs) (Yadav et al., 2019). However, research reports indicated that the prevalence rate of IM nonadherence in China ranges from 23.2 to 54.9% in RTPs (Teng et al., 2015; Xia et al., 2019; Liu et al., 2021), which was almost consistent with a worldwide incidence of 20–50% (Germani et al., 2011). Therefore, identifying the predictive factors with IM nonadherence would allow us to evaluate patients’ need for interventions to improve adherence, which could reduce the acute rejection rate and transplant graft loss to improve transplant outcomes. Numerous studies about nonadherence to IM in RTPs have indicated that demographics such as age, gender, working and socioeconomic status, educational level, and marital status were often associated with nonadherence rates (Spivey et al., 2014; Kung et al., 2017).

Several postulated theories or models have been utilized to predict and intervene nonadherence to IM in RTPs. The health belief model (HBM) model consisted of perceived susceptibility, perceived seriousness, perceived benefits, and perceived barriers, which has been widely used in many health-related behaviors (cancer, heart disease, hypertension, and renal transplant recipients) (Brown et al., 2011; Horwood et al., 2015; Zhao et al., 2015; Quast et al., 2016; Yang et al., 2016; Kung et al., 2017). An effective application of the HBM model has been conducted to predict IM nonadherence among RTPs in our transplant center, and our results indicated that perceived seriousness and perceived barriers were closely associated with IM nonadherence (Xia et al., 2019). Also, a recent study indicated that beliefs about medications were more powerful predictors to nonadherence than clinical or sociodemographic factors among patients with multiple sclerosis (Neter et al., 2021). The theory of planned behavior (TPB), as a cognitive model, also exhibited several advantages to predict a wide range of health-related behaviors (Andrew et al., 2016; Li et al., 2019; Caru et al., 2020). The TPB model only consisted of several variables (attitudes, subjective norms, perceived behavioral control, and intentions), which were avoided to include and test so many demographic and clinical factors. The validity of the TPB has been supported by different behavior domains and populations within large samples and applied successfully to predict IM nonadherence into solid organ transplant recipients (Chisholm et al., 2007; Hugon et al., 2014). Nevertheless, so far, all the predicted behavior models were usually tested independently to predict health behaviors, and few models were well suited to identify all the factors that contributed to nonadherence to a prescribed medical regimen as crucial as immunosuppressive therapy and each model had its limitations. For example, Chisholm et al. (2007) reported that the predictive capacity of the TPB could be enhanced by adding other variables such as the past behavior. Our previous study has demonstrated that the HBM could be used to predict health-related behaviors (Xia et al., 2019); however, a combined theory model to predict medication nonadherence has received little attention in RTPs. Therefore, we attempted to add the HBM variable to the TPB model to enhance the predicted power of combining the TPB and HBM model on IM nonadherence in RTPs. To the best of our knowledge, this is the first study to explore the combined effects of the TPB and HBM model on IM nonadherence in Chinese RTPs, and we hope to increase the prediction of the models on IM nonadherence and suggest some interventions to improve the IM nonadherence.

## Methods

### Study Population

This was a cross-sectional study and analyzed by structural equation modeling. The sample size should be more than 200 or 10 times of the observed variables. In our study, a total of 1,357 RTPs from transplant follow-up outpatient clinic of the Third Xiangya Hospital of Central South University were enrolled within one year from August 2019 to July 2020. The inclusion criteria were carried out as follows: 1. 18 years or older; 2. were able to communicate in Mandarin; 3. functioning renal transplant (not on dialysis); 4. qualified assessment from outpatient physician. The exclusion criteria were as follows: 1. multiple or other organ transplant recipients; 2. diagnosed with severe mental illness or cognitive impairment. All donors were derived from corresponding relatives or patients who died of cardiac diseases (Association Organ Transplantation Society of Chinese Medical, 2013).

### Study Procedures

When RTPs came for the follow-up visits in the Third Xiangya Hospital, the staff who work in the transplant follow-up outpatient clinic invited them to take part in this study. Patients who were interested in the research received an informed consent form during the consultation with the staff and completed the questionnaire in the lounge of the outpatient clinic under the supervision of the staff. Also, these four questionnaires were filled out in one time and the average time for completing these questionnaires was 25.6 min. After completing the questionnaires, all the participants received a gift (oral medicine box) to appreciate their participation. These completed questionnaires were kept by a special person to avoid information disclosure.

A written informed consent was obtained from all subjects, and this study was approved by the Ethics Committee of The Third Xiangya Hospital Central South University (No:2019-S61). At the same time, all methods were performed in accordance with the ethical guidelines and regulations of the 1975 Declaration of Helsinki.

### Instruments

A total of four questionnaires were used in this study.

#### General Data Questionnaire

Participants’ demographic characteristics were collected by this questionnaire, which included age, gender, BMI, marital status, work, religion, education, household income, transplant-related hospitalization, the time after transplantation, organ source, etc.

#### Basel Assessment of Adherence to Immunosuppressive Medications Scale

The BAASIS was a self-reported questionnaire developed by the Leuven Basel Adherence Research Group (Dobbels et al., 2010) and translated into Chinese in 2016. The previous study indicated that the BAASIS was reliable and valid to evaluate IM nonadherence in transplant recipients (Dobbels et al., 2010; De Bleser et al., 2011; Shemesh et al., 2017). We examined two dimensions of IM nonadherence in our questionnaire: implementation and discontinuation. Implementation was assessed by four questions (dose taking, drug holidays, timing deviation more than 2 h from the prescribed time, and dose reduction); overall IM nonadherence was defined as a “yes” to any of the five questions regarding to implementation or discontinuation in the last four weeks and data analysis was scored dichotomously. The BAASIS score was interpreted as acceptable in terms of reliability and validity, if the Cronbach’s *α* was greater than or equal to 0.78 (Dobbels et al., 2010; Shemesh et al., 2017).

#### Health Belief Model

The researcher developed this study questionnaire based on the Rosenstock’s health belief model and the Chinese version of HBM has been validated in Chinese hypertension patients (Zhao et al., 2015). It constructed perceived susceptibility regarding self-awareness of infection and adverse reactions of the medication (three items); perceived seriousness regarding individual awareness of impact of rejection, infection and other complications, and their survival (four items); perceived benefits of adherence to treatment with IM regarding subjective beliefs whether better adherence lowers the possibility of complications (four items); and perceived barriers to adherence regarding the adverse effects of medication and some living conflicts (four items). Each item on the IM belief questionnaire was structured using a 5-point Likert scale ranging from 1 (strongly disagree) to 5 (strongly agree). As for the perceived barriers, the scaling was opposite the other constructs. The reliability of each questionnaire was tested using Cronbach’s *α*. The range of Cronbach’s *α* among Chinese patients was 0.77–0.90 (Zhao et al., 2015).

#### TPB Questionnaire

We adapted the TPB questionnaire initially developed and validated in RTPs by Chisholm et al. (2007) and the Chinese version of TPB has been validated in Chinese RTPs and dialysis patients (Du et al., 2018; Li C. et al., 2019). The questionnaire explored attitudes (twelve items), subjective norms (five items), perceived behavioral control (two items), past behavior (two items), and intentions (two items). Evidence for the reliability and predictive validity of the TPB model have been supported by numerous studies (Andrew et al., 2016; Li A. S. W. et al., 2019; Caru et al., 2020). The reliability of each questionnaire was tested using Cronbach’s *α*, and the range of Cronbach’s *α* among the Chinese RTPs was 0.606–0.855 (Du et al., 2018).

#### Data Analysis

All statistical analyses were performed using the SPSS version 20.0 (SPSS Inc., Chicago, IL, United States). The sociodemographic characteristics were analyzed using descriptive statistics. The categorical variables were summarized by numbers or percentages. Relationships among sociodemographic characteristics, IM beliefs, and IM nonadherence were analyzed using the *chi-square test* and binary logistic regression analysis. The TPB structural equation was run by Amos 21.0 (Analysis of Moment Structures, IBM, Armonk, NY, United States). Spearman’s rank correlation coefficient was employed to measure associations between categorical TPB and HBM variables and medication nonadherence. A *p* value <0.05 (two-tailed) was considered statistically significance.

## Results

### Characteristics of the Participants

**FIGURE 1:**
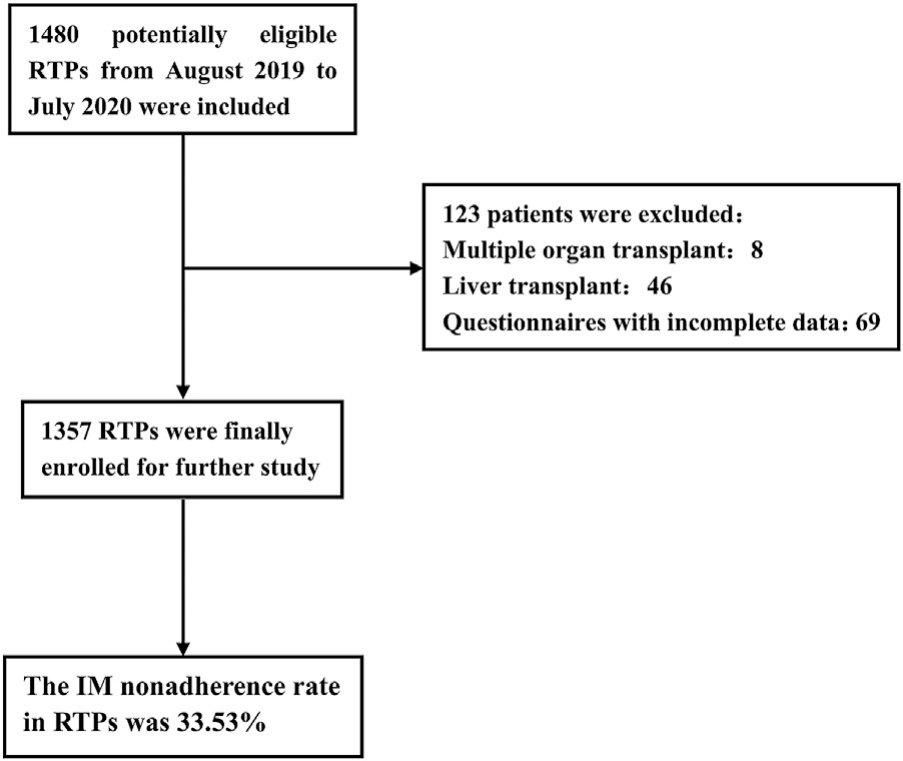
The study flowchart.

In this study, a total of 1,480 RTPs in the follow-up outpatient clinic were invited and 1,357 valid questionnaires were enrolled for further study (Figure 1). The sociodemographic characteristics of the participants are summarized in Table 1. The mean age and BMI were 41.25 years and 22.75 kg/m^2^, respectively. 67.80% of the participants were male, 61.02% of them were married, 48.71% of them had a higher education level than undergraduate, and 93.81% of them lacked religion. Meanwhile, we found that only 42.08% of them were still working. Approximately 91.82% of patients received grafts from deceased donors. About half of the participants had preoperative drinking history and 56.67% of them were hospitalized after transplantation.

**TABLE 1:** Sociodemographic characteristics of RTPs (n = 1,357).

variables		N	%
Age (y)	18–20	22	1.62
	21–30	160	11.79
	31–40	478	35.22
	41–50	458	33.75
	>51	239	17.61
Gender	Male	920	67.80
	Female	437	32.20
BMI	<18.5	165	12.16
	18.5–24	812	59.84
	24–28	302	22.25
	>28	78	5.75
Work	No	786	57.92
	Yes	571	42.08
Religion	No	1,273	93.81
	Yes	84	6.19
Education	≦Secondary school	259	19.09
	High school	437	32.20
	Post/undergraduate	661	48.71
Marital status	Unmarried	333	24.54
	Married	828	61.02
	Divorced/Widowed	196	14.44
Preoperative drinking history	No	689	50.77
	Yes	668	49.23
Time after transplantation (month)	≦6	222	16.36
		285	21.00
	12–36	477	35.15
	>36	373	27.49
Transplant-related hospitalization	No	588	43.33
	Yes	769	56.67
Organ source	DCD	1,246	91.82
	Relative donor	111	8.18
Household income (RMB)	≦3,000	466	34.34
	3,000–5,000	363	26.75
	>5,000	528	38.91

RMB: Chinese Yuan Renminbi; DCD: organ donation after death.

### The IM Nonadherence in the Participants

The overall IM nonadherence rate was 33.53% based on the BAASIS assessment. In detail, our results indicated that 31.10% of RTPs happened to the item of “In the last four weeks the IM was taken more than 2 h before or after the prescribed dosing time,” which was the most commonly occurred. Meanwhile, we found that 19.81% of RTPs missed one dose and 10.02% of RTPs skipped two or more doses. The percentage of RTPs who altered the prescribed amount and completely stopped the intake of IM was 7.15 and 1.47%, respectively (Table 2).

**TABLE 2:** Adherence to IM measured by BAASIS.

items		N	%	Nonadherence rate (%)
Do you remember missing one dose of your IM in the last 4 weeks?	None	1,088	80.18	19.81
	1 time	201	14.81	
	2 times	58	4.27	
	3 times	10	0.74	
	4 times	0	0.00	
	>4 times	0	0.00	
Do you remember having skipped 2 or more doses of your IM in row in the last 4 weeks?	None	1,221	89.98	10.02
	1 time	121	8.92	
	2 times	15	1.11	
	3 times	0	0.00	
	4 times	0	0.00	
	>4 times	0	0.00	
Do you remember having taken your IM more than 2 h before or after the recommended dosing time in the last 4 weeks?	None	935	68.90	31.10
	1 time	242	17.83	
	2 times	136	10.02	
	3 times	22	1.62	
	4 times	12	0.89	
	>4 times	10	0.74	
Have you altered the prescribed amount (e.g., taken more or fewer pills or changed your dose) of your IM during the last 4 weeks without your doctor telling you to do so?	None	1,260	92.85	7.15
	1 time	85	6.26	
	2 times	12	0.89	
	3 times	0	0.00	
	4 times	0	0.00	
	>4 times	0	0.00	
Have you stopped taking your IM completely within the last year without your doctor telling you to do so?	Yes	20	1.47	1.47
	No	1,337	98.53	

The overall nonadherence rate was 33.53% (455/1,357).

### Predictors of Nonadherence in the Univariate and Logistic Regression Analyses

Table 3 showed the univariate analyses of the relationship between all sociodemographic variables and IM nonadherence. All sociodemographic variables, except for transplant-related hospitalization, manifested statistical significance. Furthermore, our logistic regression analysis revealed that a variety of variables, such as marital status, religion household income, preoperative drinking history, posttransplant time, each dimension of HBM, attitudes, past behavior, perceived behavioral control, and intentions, were independent predictive nonadherence factors to IM (Table 4).

**TABLE 3:** Univariate analysis of the association between sociodemographic characteristics and IM adherence among RTPs.

variables		Nonadherence	Adherence	Χ^2^	*P*
Age (y)	18–20	0	22	58.36	0.000
	21–30	82	78		
	31–40	173	305		
	41–50	155	303		
	>51	45	194		
Gender	Male	383	537	84.11	0.000
	Female	72	365		
BMI	<18.5	29	136	34.29	0.000
	18.5–24	263	549		
	24–28	130	172		
	>28	33	45		
Work	No	109	462	92.24	0.000
	Yes	16	139		
Religion	No	453	820	38.98	0.000
	Yes	2	82		
Education	≦Secondary school	74	185	11.52	0.003
	High school	130	307		
	Post/undergraduate	251	410		
Marital status	Unmarried	247	86	573.71	0.000
	Married	78	750		
	Divorced/Widowed	130	66		
Preoperative drinking history	No	51	638	428.72	0.000
	Yes	404	264		
Organ source	DCD	406	840	6.11	0.013
	Relative donor	49	62		
Time after transplantation (month)	≦6	103	119	203.69	0.000
	6–12	126	159		
	12–36	42	435		
	≧36	184	189		
Household income (RMB)	≦3,000	202	264	108.50	0.000
	3,000–5,000	42	321		
	>5,000	211	317		
Transplant-related hospitalization	No	186	402	1.68	0.195
	Yes	269	500		

RMB: Chinese Yuan Renminbi; DCD: organ donation after death.

**TABLE 4:** Binary logistic regression analysis of the association between sociodemographic characteristics and IM adherence among RTPs.

variable	B	S.e,	Or	95% CI		*p*
Marital status (unmarried)						0.000
Married	−1.960	0.503	0.141	0.052	0.378	0.000
Divorced/Widowed	0.559	0.514	1.748	0.639	4.786	0.277
Religion	1.803	0.906	6.065	1.027	35.817	0.047
Household income (≦3,000)						0.002
3,000–5,000	−1.145	0.509	0.318	0.117	0.863	0.024
>5,000	0.759	0.411	2.136	0.955	4.776	0.065
Time after transplantation (month) (≦6)						0.000
6–12	−2.229	0.667	0.108	0.029	0.398	0.001
12–36	−1.778	0.661	0.169	0.046	0.618	0.007
≧36	−0.055	0.61	0.947	0.286	3.128	0.928
Preoperative drinking history	1.537	0.485	4.651	1.799	12.023	0.002
HBM-perceived susceptibility	−0.328	0.1	0.72	0.592	0.875	0.001
HBM-perceived seriousness	−0.44	0.112	0.644	0.517	0.802	0.000
HBM-perceived benefits	0.888	0.152	2.431	1.805	3.274	0.000
HBM-perceived barriers	−0.505	0.075	0.603	0.521	0.699	0.000
TPB-attitudes	−0.168	0.038	0.846	0.785	0.91	0.000
TPB-perceived behavioral control	−0.648	0.115	0.523	0.418	0.656	0.000
TPB-intentions	−1.111	0.191	0.329	0.227	0.478	0.000
TPB-past behavior	0.502	0.225	1.652	1.064	2.566	0.025
Constant	19.411	4.735	2.693E+8			0.000

R^2^ = 89.7%.

### TPB and HBM Measurements

In our study, each dimension of TPB and HBM questionnaires were significantly correlated with IM nonadherence (*p* < 0.05), but the dimension of HBM had a higher interrelationship to IM nonadherence than that of TPB (Table 5). In addition, perceived barriers to adherence exhibited significantly negative correlation with adherence (r = −0.743, *p* < 0.01).

**TABLE 5:** Interrelationship between each dimension of two models and IM adherence.

	Adherence	Perceived susceptibility	Perceived seriousness	Perceived benefits	Perceived barriers	Hbm	Attitudes	Subjective norms	Perceived behavioral control	Intentions	Past behavior
Adherence	1										
Perceived susceptibility	−0.516**	1									
Perceived seriousness	−0.507**	0.408**	1								
Perceived benefits	0.084**	0.085**	0.278**	1							
Perceived barriers	−0.743**	0.483**	0.511**	−0.003	1						
HBM	−0.702**	0.695**	0.676**	0.337**	0.873**	1					
Attitudes	−0.234**	0.175**	0.180**	0.175**	0.347**	0.338**	1				
Subjective norms	-−0.080**	0.155**	0.028	0.156**	0.145**	0.204**	0.130**	1			
Perceived behavioral control	−0.201**	−0.009	0.105**	0.329**	0.061*	0.157**	0.025	0.042	1		
Intentions	−0.151**	0.084**	−0.011	0.052	0.025	0.056*	0.219**	0.317**	0.091**	1	
Past behavior	−0.058*	0.041	0.111**	0.089**	0.153**	0.141**	0.209**	−0.109**	−0.055*	−0.074**	1

*p < 0.05 **p < 0.01.

### Combined Theory Model Improved the Prediction of IM Nonadherence Significantly

**FIGURE 2:**
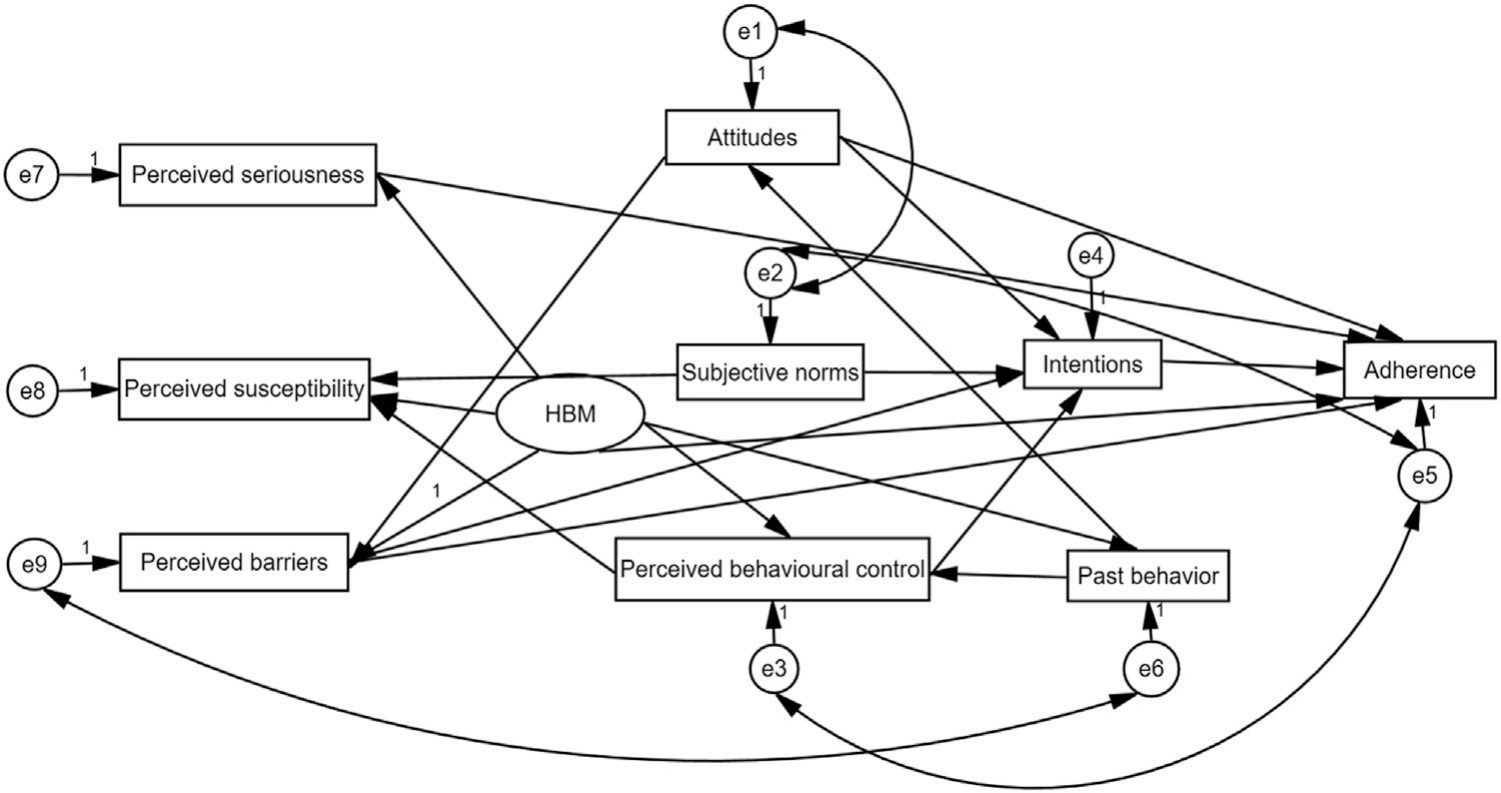
Summary of regression results of the combined theory model dimensions.

In the current study, TPB alone, as a superior cognitive model, only explained 2% of variance in nonadherence to IM. However, combining the TPB and HBM model could explain 52% of variance in nonadherence to IM (Figure 2). Therefore, it was clear that incorporation of the HBM model was made it possible to improve the prediction capacity of the TPB model. In detail, perceived barriers, attitudes, and HBM contributed to the overall effect to IM nonadherence. HBM had the highest coefficient of effect (−0.620) and perceived barriers was second (0.284). Perceived seriousness and intentions influenced IM nonadherence directly with coefficient of effect 0.256 and −0.126. Past behavior (−0.044), subjective norms (−0.035), and perceived behavioral control (−0.009) influenced IM nonadherence indirectly *via* mediators of intentions (Table 6). Also, the model fitted the data well, and the CMIN/DF, RMSEA, GFI, CFI, and IFI values of the combined model were 2.469, 0.033, 0.995, 0.992, and 0.992, respectively.

**TABLE 6:** Effect of each latent variable of the structural equation model on medication intentions and adherence.

Latent variables	Direct effect	Indirect effect	Total effect
HBM→ intentions	—	−0.096	−0.096
Past behavior→ intentions	—	0.049	0.049
Attitudes→ intentions	0.317	−0.033	0.284
Perceived barriers →intentions	−0.149	—	−0.149
Perceived behavioral control →intentions	0.068	—	0.068
Subjective norms→ intentions	0.278	—	0.278
HBM→ adherence	−0.562	−0.058	−0.620
Past behavior→ adherence	—	−0.044	−0.044
Attitudes→ adherence	−.125	−0.102	−0.227
Perceived barriers →adherence	−0.303	0.019	−0.284
Perceived behavioral control →adherence	—	−0.009	−0.009
Subjective norms→ adherence	—	−0.035	−0.035
Perceived seriousness →adherence	0.256	—	0.256
Intentions →adherence	−0.126	—	−0.126

## Discussion

Our article initiated the first study of the incorporation between TBP variables and the HBM model in IM nonadherence in Chinese RTPs. Also, the purpose of our work was to conduct the combined model to determine the barriers to IM nonadherence and provide some new insights on how to improve the IM adherence among the RTPs.

In the current study, the IM nonadherence of the patients was 33.53%, which was in accordance with previously reported 5–50% range of nonadherence rate in renal transplantation (Leendertse et al., 2008; Germani et al., 2011). Forgetfulness was one of the major reasons for nonadherence to medications in other clinical settings such as HIV (Morowatisharifabad et al., 2019). Better drug reminder also effectively reduced the rate of nonadherence.

The binary logistic regression analysis suggested that several sociodemographic characteristics such as marital status, household income, preoperative drinking history, the time after transplantation, and religion were independent predictive factors of IM nonadherence. Our study indicated that distinct marital statuses exhibit different capacity of IM nonadherence; married recipients were more likely to adhere to IM than to single or divorced recipients. Additionally, findings from other studies have revealed that the family member–based supervised therapy used in patients with hypertension and type 2 diabetes may have positive effects on patient IM nonadherence (Mayberry and Osborn, 2012; Huang et al., 2014; Shen et al., 2017).

Meanwhile, family income had negative effect on IM nonadherence. Although a Brazilian study reported that higher family income was the only factor that was associated with immunosuppressive nonadherence because of lower income recipients benefiting from better access to care and coverage of health-care costs after transplantation (Marsicano et al., 2015), higher family income could support transplant recipients to pay for their IM, check the IM concentration, and improve the quality of life in China. Also, the life quality manifested a direct effect on medication adherence in Asian RTPs (Ganjali et al., 2019). In addition, religion issues also displayed a considerable influence on positive attitudes toward transplantation (Messina, 2015; Kobus et al., 2016). Religions like Christianity, Islam, Judaism, Buddhism, or other denominations provided guidance and principles for their behavior and thinking in these areas. Therefore, the recipients with religions may have some special etiquettes to limit the use of IM.

Moreover, our result showed that the recipients within half to one year were more likely adherent to IM. Interestingly, the recipients with more than three years did not show significant correlation with adherence to IM. Many studies reported that RTPs believed their allografts would become relatively histocompatible over a longer time (Chisholm et al., 2007; Zhao et al., 2017). In the present study, one of the plausible reasons was that the patients were more appreciative of their allografts and cautious in the early postoperation. Also, the more the time passes, the less the appreciation they have. Meanwhile, doctor’s advices and patients’ positive attitudes about the usage of immunosuppressive drugs for allograft might be diluted when time passes.

Finally, RTPs with preoperative drinking history were prone to IM nonadherence and studies in alcoholism have highlighted the impairments of affecting episodic memory as well as semantic and cognitive procedural learning (Pitel et al., 2007; Le Berre et al., 2010; Noël et al., 2012; Le Berre et al., 2017). So patients with preoperative drinking history tend to forget to take IM. From the above results, important strategies for intervention are needed to be utilized to minimize the barriers and improve the adherence to IM.

In the present study, TPB only explained 2% of variance in adherence to IM. Based on our previous empirical and theoretical work (Xia et al., 2019), IM beliefs were closely associated with IM nonadherence in Chinese RTPs. Our result showed that combining the TPB and HBM model could explain 52% of variance in IM adherence as well as all variables except for the subjective norms were independent predictive factors of IM nonadherence according to the logistic analysis. Several studies have noted that due to variations in the behavior under investigation, it should not be expected that all TPB factors (attitudes, subjective norms, and perceived behavioral control) will always be significant and stronger support for some components of the TPB than others, with subjective norms often emerging as the weakest predictor (Chisholm et al., 2007). Our results indicated that though the small items of the TPB model showed some advantages in predicting a wide range of health-related behaviors, it did not exhibit superiority in our large sample study. Based on our previous empirical and theoretical work (Xia et al., 2019), we integrated the HBM variables into the TPB structural equation model and found that the combined model has the ability to improve the predictive capacity of IM nonadherence than that in any single model. Therefore, we proposed that the combination of TPB and HBM exhibits more advantages in predicting IM nonadherence in studies with large samples.

In the structural equation model, we discovered that attitudes act as a salient predictor to IM intentions (0.284), both directly and indirectly. Chisholm MA et al reported that those RTPs who were particularly at risk might have a history of nonadherence to medical advice, especially when they had negative attitude about IM adherence (Chisholm et al., 2007; Hugon et al., 2014). An individual's perception of the "benefits and harms" of the target behavior is crucial to the behavioral attitude. Thus, important interventions to improve and strengthen attitudes about IM benefits were needed to promote the IM adherence. Notably, subjective norms were second only to attitudes in predicting IM intentions (0.278). In other words, the greater the influence of other individual or group giving to RTPs in IM adherence, the stronger the intentions to take medication. Therefore, medical staff should not only provide positive psychological interventions and social support to RTPs but also enhance their family’s awareness of taking IM regularly and timely.

In our study, HBM contributed the overall effect to IM nonadherence with the highest coefficient of effect and perceived barriers could play a vital influence on IM nonadherence. Our result was consistent with some previous studies showing that perceived barriers tend to be the strongest predictor of compliance (Wang et al., 2006; Carpenter, 2010). However, a recent study provided some different evidence that perceived susceptibility to rejection and perceived benefits of treatment were identified as major predictors of IM nonadherence (Kung et al., 2017). This difference might be due to different sample sizes in these studies. Nevertheless, all studies confirmed that health-related beliefs measured by HBM is an effective predictor of IM nonadherence. Based on our results, future educational programs should focus on the seriousness of not taking IM for Chinese RTPs. Some perceiving barriers, such as forgetting to take medicine and conflicts with social events, were similar to other reports. A recent study about hypertension population reported that forgetting was the most common reason for causing difficulty in taking medication (Yang et al., 2016). Understanding the barriers to optimal adherence provided information that could be used to better target interventions by doctors or health-care professionals. According to the present results, some effective methods, such as electronic medication devices with reminder and record functions, could play important roles in enhancing RTPs’ adherence. Our study also found that all variables of the TPB model did not exhibit sufficient effects on IM nonadherence, which might be one explanation of why TPB only explained 2% of variance in IM nonadherence. Until now, IM nonadherence could be predicted by several models, each having advantages and disadvantages, but none was considered as the gold standard. So we hoped that combination of the TPB and HBM model could make up for their shortcomings and provide much more interventions to improve transplant outcomes in clinical applications.

The most important aspect of this study included using the combined model and representing the larger sample size. Admittedly, the present study still had some limitations. One limitation was the potential bias, which was considered as follows: 1) A prestudy sample size calculation was absent. Hence, the findings of this study should be interpreted with caution. This is because the absence of sample size calculation may lead to systematic errors. 2) The adherence questionnaire used in this study was based on self-report, hence there could be recall bias. 3) Randomization was not employed in the selection of participants, hence the way the sample was obtained could introduce selection bias. Another limitation was that our study lacked effective external validity at present because of the COVID-19. In the future, multicenter studies with larger samples of different age-groups and some objective measures of IM nonadherence are needed to confirm our conclusions.

## Conclusion

Our study, to our knowledge, is the first to explore the combined effect of the TPB and HBM model on IM nonadherence in Chinese RTPs, which could significantly improve the predictive capacity of any single model in IM nonadherence. Important implications for intervention can be drawn from our results. The logistic regression analysis results suggested that medical staff should focus on the RTPs with single or divorced status, families with low income, long time course after renal transplantation, and preoperative drinking history. Moreover, the combined model implied that encouraging RTPs’ willingness toward IM and enhancing their families’ awareness of taking IM regularly and timely were great ways to improve IM adherence. Meanwhile, future interventions should be conducted to increase perceived seriousness and to reduce perceived barriers for taking IM, which will effectively increase IM adherence rates and reduce the rejection risk to improve transplant outcomes. Future multicenter validation about the prediction and prevention of IM nonadherence are needed to confirm our conclusions, which would make more RTPs benefit.

## Data Availability

The raw data supporting the conclusions of this article will be made available by the authors, without undue reservation.

## Abbreviations

IM, immunosuppressive medication; TPB, theory of planned behavior; HBM, health belief model; RTPs, renal transplant patients; BAASIS, basal assessment of adherence to immunosuppressive medications scale.
